# 401. Natural History of Shedding and Household Transmission of Severe Acute Respiratory Syndrome Coronavirus 2 Using Intensive High-Resolution Sampling

**DOI:** 10.1093/ofid/ofab466.602

**Published:** 2021-12-04

**Authors:** Jonathan Altamirano, Prasanthi Govindarajan, Andra Blomkalns, Sean Leary, India Robinson, Leanne Chun, Nuzhat Shaikh, Grace Tam, Marcela Lopez, Makeda Robinson, Yuan J Carrington, Monique De Araujo, Katharine Walter, Jason Andrews, Catherine Hogan, Benjamin A Pinksy, Yvonne A Maldonado

**Affiliations:** 1 Stanford University School of Medicine, Stanford, CA; 2 Stanford University, Stanford, CA

## Abstract

**Background:**

In order to mitigate the spread of SARS-CoV-2 and the COVID-19 pandemic, public health officials have recommended self-isolation, self-quarantine of exposed household contacts (HHC), and mask use to limit viral spread within households and communities. While household transmission of SARS-CoV-2 is common, risk factors for HHC transmission are poorly understood.

**Methods:**

In this prospective cohort study, we enrolled 37 households with at least one reverse transcription polymerase chain reaction-confirmed (RT-PCR) COVID-19 index case from March 2020 - March 2021, in order to calculate secondary attack rates (SAR) and define risk factors for secondary infections. Participants were tested daily for SARS-CoV-2 via RT-PCR, using self-collected lower nasal samples. Households were followed until all members tested negative for seven consecutive days. We collected demographics, medical conditions, relationship to index case, and socioeconomic indicators. Subgroup data analysis was conducted and stratified by positivity status.

**Results:**

Of 99 enrolled participants, 37 were index cases and 62 were household contacts (HHC), of whom 25 HHC were infected (40.3%). Secondary attack rate (SAR) was highest among adults caring for a parent (n=4/4, 100%) and parents of index cases (5/10, 50%). Households whose income came from service work had greater risk of transmission compared to households whose primary income was technology (n=5/7; 71.4% vs 3/8; 37.5% respectively). Pediatric contacts were at lower risk of infection when compared to adult contacts (n=5/18, 27.8% vs n=20/44, 45.5% respectively).

**Conclusion:**

This study suggests that household transmission represents a key source of community-based infection of SARS-CoV-2. Allocating resources for education/training regarding prevention among infected individuals and their close contacts will be critical for control of future outbreaks of SARS-CoV-2.

**Disclosures:**

**All Authors**: No reported disclosures

